# An optimized SPE-LC-MS/MS method for antibiotics residue analysis in ground, surface and treated water samples by response surface methodology- central composite design

**DOI:** 10.1186/s40201-017-0282-2

**Published:** 2017-10-17

**Authors:** Roya Mirzaei, Masoud Yunesian, Simin Nasseri, Mitra Gholami, Esfandiyar Jalilzadeh, Shahram Shoeibi, Hooshang Shafieyan Bidshahi, Alireza Mesdaghinia

**Affiliations:** 1 Center for Water Quality Research (CWQR), Institute for Environmental Research (IER), Tehran University of Medical Sciences, 8th floor, Gol Building, North Karegar St., Enghelab Sq, Tehran, Iran; 2 Department of Environmental Health Engineering, School of Public Health, Tehran University of Medical Sciences, Tehran, Iran; 3 Center for Air Pollution Research (CAPR), Institute for Environmental Research (IER), Tehran University of Medical Sciences, Tehran, Iran; 4 Department of Environmental Health Engineering, School of Public Health, Iran University of Medical Sciences, Tehran, Iran; 5 Water and Wastewater Company, Department of Water and Wastewater Quality Control Laboratory, Tehran, Iran; 6 Food and Drug Reference Control Laboratories Center, Food and Drug Organization, Ministry of Health & Medical Education, Tehran, Iran

**Keywords:** LC/MS/MS, Solid phase extraction, Response surface methodology, β-lactam antibiotics, Cephalosporins

## Abstract

**Background:**

Antibiotic residues are being constantly identified in environmental waters at low concentration. Growing concern has been expressed over the adverse environmental and human health effects even at low concentration. Hence, it is crucial to develop a multi-residues analytical method for antibiotics to generate a considerable dataset which are necessary in the assessment of aquatic toxicity of environmental waters for aquatic organisms and human health. This work aimed to develop a reliable and sensitive multi-residue method based on high performance liquid chromatography coupled with quadrupole-linear ion trap tandem mass spectrometry (HPLC-MS-MS). The method was optimized and validated for simultaneous determination of four classes of antibiotics including, β-lactam, macrolide, fluoroquinolone and nitro-imidazole in treated, ground and surface water matrices.

**Methods:**

In order to optimize the solid phase extraction process, main parameters influencing the extraction process including, pH, the volume of elution solvent and the amount of Na_4_EDTA were evaluated. The optimization of extraction process was carried out by response surface methodology using central composite design. Analysis of variance was performed for nine target antibiotics using response surface methodology.

**Results:**

The extraction recoveries were found to be sensitive to the independent variables of pH, the volume of elution solvent and the amount of Na_4_EDTA. The extraction process was pH-dependent and pH was a significant model term in the extraction process of all target antibiotics. Method validation was performed in optimum operation conditions in which the recoveries were obtained in the range of 50–117% for seven antibiotics in spiked treated and ground water samples and for six antibiotics in spiked river water samples. Method validation parameters in terms of method detection limit were obtained in the range of 1–10 ng/L in treated water, 0.8–10 ng/L in the ground water and 0.8–25 ng/L in river water, linearity varied from 0.95 to 0.99 and repeatability in term of relative standard deviation values was achieved less than 10% with the exception for metronidazole and ceftriaxone. The developed method was applied to the analysis of target antibiotics in treated, ground and surface water samples.

**Conclusions:**

Target antibiotics were analyzed in different water matrices including treated, ground and river water. Seven out of nine antibiotics were detected in Kan River and Firozabad Ditch water samples, although none of them were detected in treated water and ground water samples.

**Electronic supplementary material:**

The online version of this article (10.1186/s40201-017-0282-2) contains supplementary material, which is available to authorized users.

## Background

Antibiotics are one of the most important groups of pharmaceuticals used in human and veterinary medicine extensively. Concerns have been growing worldwide about their occurrence in environmental waters. Considering that they are metabolized to some extent after administration, unmetabolized active compounds are excreted in urine (generally 55–80%) and feces as a mixture of metabolites or conjugated compounds [1]. Accordingly, they can enter water bodies through effluent of urban wastewater treatment plants (UWWTP), hospital and pharmaceutical treatment plants due to their removal inefficiently by conventional systems [2, 3]. Furthermore, the release of effluent into water bodies, which can serve as a source for drinking water production facilities, is truly significant because the quality of people’s life who live in downstream areas can be affected by antibiotic residues [4–6]. Thus, it is significant to establish a sensitive and reliable analytical method to determine the amount of multi-residue of antibiotics or other pharmaceutical active compounds (PhACs) at the concentration range of ng/L and low μg/L in water bodies.

Many researchers have developed methods to find a board range of antibiotics in various water matrices [7–13]. All these methods are based on liquid chromatography coupled with tandem mass spectrometry. In these methodologies, many parameters related to chromatographic conditions such as the ionization response of electrospray ionization mass spectrometry (ESI-MS), and the extraction process which has been optimized individually in different levels. However, the interaction between variables hadn’t been taken into account statistically.

Therefore, a systematic approach such as an experimental design to optimize the extraction process is highly essential. Furthermore, diverse physicochemical properties of pharmaceutical compounds, such as antibiotics, make them display different extraction efficiencies after extraction process and analysis. These variables include pH, the amount of Na_4_EDTA and the volume of elution solvent. Traditional procedures of optimization like (one-variable-at-a-time) are time-consuming and costly, while they fail to show interactions among the parameters. On the other hand, many researchers worked under different conditions, including neutral and acidic condition, various amounts chelating agent and different volumes of elution solvent. Hence similar and contrasting recoveries have been reported [8, 14, 15]. In order to optimize significant parameters in an extraction process, multivariate optimization strategies such as Response Surface Methodology (RSM) based on experimental design was applied as a widely used approach for evaluating the main effects and variable interactions within at least a number of runs [16, 17]. RSM, which is based on quadratic polynomial equation, is considered as the most useful method to predict the optimum parameters in sample preparation [16].

Since six out of nine target antibiotics in this study were β-lactam and cephalosporin, the physicochemical properties and chromatographic behavior of β-lactam and cephalosporin have been particularly emphasized in this optimization method. According to previous studies, β-lactam antibiotics (BLs) are widely used due to their anti-microbial activities against gram-positive and gram-negative bacteria. Moreover their therapeutic and preventive properties, these compounds have been widely used as growth promoters in livestock animals [5]. The identification of BL compounds has been reported by a few studies in different water matrices, including wastewater treatment plant effluent [8, 18] and hospital wastewater [15], as well as ground, surface and drinking water [12, 14, 19, 20]. However, these compounds are considered to be unstable in water matrices due to their chemical structures, so there are many restrictions to their simultaneous analysis through multi-residue methodologies. In addition, optimum conditions for their analysis could not be achieved and thus low recoveries were obtained for β-lactam antibiotics, including amoxicillin and penicillin [8, 21].

In present work, a reliable extraction method was developed for simultaneous analysis of two β-lactam antibiotics (amoxicillin and penicillin G), three cephalosporins (cefixime, cephalexin, and ceftriaxone), two macrolides (azithromycin, erythromycin), one nitro-imidazole (metronidazole), and one fluoroquinolone (ciprofloxacin) in HPLC grade water. The effects of the variables (amount of Na_4_EDTA, pH and the volume of elution solvent) were investigated and optimized using the Central Composite Design (CCD) combined with Response Surface Methodology (RSM), followed by the Desirability Function (DF) in order to maximize the criteria of the response. Then in optimum conditions, the method was validated in treated, ground and surface water. Finally, the optimized method was applied to natural water samples.

## Methods

### Chemicals and samples

All antibiotic compounds including amoxicillin and penicillin G, cephalexin, ceftriaxone, cefixime, ciprofloxacin, azithromycin, erythromycin, metronidazole were of high purity grade (>99%) and purchased from Sigma-Aldrich (Steinham, Germany) which are listed in Table 1. All solvents used, including the HPLC grade methanol (MeOH), acetonitrile (ACN), HPLC grade water, hydrochloric acid 37% and formic acid 98% were purchased from Merck (Darmstadt, Germany). Tetrasodium Ethylenediaminetetraacetate (Na_4_EDTA) was obtained from Sigma-Aldrich (Steinham, Germany). Nitrogen for drying was of high purity grade (>99/9998%) and was supplied by Tehran Gas Co. (Tehran, Iran). Isotopically labeled compounds used as internal standards were ciprofloxacin-d8, azithromycin-d3, metronidazole-d4, and cefuroxime-d3, which were purchased from Santa Cruz Biotechnology (California, US) (Table 1). Oasis HLB cartridges (6 mL, 500 mg) were supplied by Waters (Milford, USA). Glass fiber filters (GF/F, pore size 1 μm) and nylon membrane filters (0.45 μm) were supplied by Merck Millipore (Darmstadt, Germany).

**Table 1:** Target antibiotics organized by their therapeutical groups, structures, molecular weight (MW), PKa values and their assigned internal standard with their characteristics

Chemical Groups	compound	Chemical formula	MW	PKa	Corresponding internal standard	CAT number of internal standard	Chemical formula for internal standards
Β-lactam	Amoxicillin	C16H19N3O5S	365.4 g/mol	3.32	Cefuroxime-d3	sc-217,864	C16H13D3N4O8S
	Penicillin G	C16H18N2O4S	334.4 g/mol	2.74	Cefuroxime-d3	sc-217,864	C16H13D3N4O8S
Cephalosporin	Cephalexin	C16H17N3O4S	347.39 g/mol	4.5	Cefuroxime-d3	sc-217,864	C16H13D3N4O8S
	Ceftriaxone	C18H18N8O7S3	554.58 g/mol	3.19	Cefuroxime-d3	sc-217,864	C16H13D3N4O8S
	cefixime	C16H15N5O7S2	453.452 g/mol	3.45	Cefuroxime-d3	sc-217,864	C16H13D3N4O8S
Fluoroquinolones	Ciprofloxacin	C17H18FN3O3	331.346 g/mol	5.76	Ciprofloxacin-d8	sc-217,902	C17H10D8FN3O3•HCl
Macrolide	Azithromycin	C38H72N2O12	748.984 g/mol	8.74	Azithromycin-d3	sc-217,686	C38H69D3N2O12
	Erythromycin	C37H67NO13	733.93 g/mol	8.88	Azithromycin-d3	sc-217,686	C38H69D3N2O12
Nitro imidazole antibiotics	Metronidazole	C6H9N3O3	171.15 g/mol	15.44	Metronidazole-d4	sc-217,686	C6H5D4N3O3

### Sample collection

One-third of total water consumption in Tehran was supplied by ground water resources. The water withdrawn from all groundwater wells, after quality control, is directed to reservoirs. After disinfection, the water is transferred to the distribution and consumption network. Thirteen water wells representing different districts of Tehran were selected randomly. At each site, water samples were collected as close to the wellhead as possible before chlorination and after the field parameters were stabilized (electrical conductivity, temperature, pH, dissolved oxygen) in the volume flow. Thirteen drinking water wells were sampled, one grab sample in each sampling event. A total of 39 water well samples were collected during the sampling campaign.

Raw water and treated water samples were collected from five different drinking water treatment plants (DWTPs) in Tehran. They were major drinking water suppliers in Tehran during three sampling campaigns on June 13, 2016 (wet season), July 9, 2016 (dry season), and August 2, 2016 (dry season). Two rivers (Kan River and Firozabad Ditch) in Tehran province river basin were the subjects of the study. Along the way, domestic and industrial wastewater is released into the stream, which can cause chemical and microbiological contamination in agricultural fields and gardens which are irrigated by this creek in Varamin and Ray areas in the south of Tehran. Two sampling sites were selected on the rivers. River water samples were collected from each sampling site in duplicate on each sampling event. One liter bottles (polypropylene containers) were used for taking grab samples. Sodium thiosulfate (20 mg/L) was added to the treated water sample bottles as a quenching agent [22]. All samples were carried to the library in a cool box at 4 °C within 6–12 h.

### Preparation of standard solutions and samples

Stock standard solutions were prepared individually in a concentration of 1 mg/mL, by dissolving 10 mg of solid reference of each standard in 10 mL of proper solvent. Penicillin and cephalosporins were dissolved in HPLC grade water because of their stability in HPLC water [8]. Fluoroquinolone antibiotics including ciprofloxacin take a long time to dissolve in pure methanol, so the compound was dissolved in MeOH/HPLC water (50:50, *v*/v) with having 0.2% hydrochloric acid [22]. The rest of the target analytes were dissolved in methanol. Stock standard solutions were stored at −20 °C and they were renewed monthly due to their instability [8]. Fresh stock standard solutions for penicillin and cephalosporin were prepared monthly and stock standard solution for the rest of compounds were prepared every three months. Afterward, working standard solutions were prepared in MeOH/HPLC water (50:50, *v*/v) in gradient dilution and they were refreshed before each run. A mixture of isotopically labeled internal standards was prepared in methanol and further dilution was also prepared in MeOH /HPLC water (50:50, v/v).

Three kinds of samples were prepared, the first ones were samples for optimization procedure, Individual standard solutions of antibiotics at a concentration of 1 mg/mL were prepared in appropriate solvents and then a mixture working standard solution of antibiotics at a concentration of 1 μg/mL was prepared in MeOH /HPLC water (50:50, v: v). Afterward, the significant extraction parameters were studied and optimized by spiking 80 ng/L of the mixture solution of antibiotics in 100 mL of ultrapure water. The recoveries of antibiotics are presented in Table S1 (see Additional file 1). After optimal conditions were obtained, the second parts of samples were prepared to construct the linear rang 500 mL of ground, treated and river water samples were spiked by stock standard solution of each antibiotic individually in the range of concentration 0.5–500 ng/L and fixed amount of the internal standard mixture in the concentration of 80 ng/L then extracted by solid phase extraction. Finally, the third ones were the samples which prepared for method validation. 500 mL of ground, treated and river water samples were spiked by stock standard solution of a mixture of antibiotic at a concentration of 100 ng/L then extracted and analyzed.

### Solid phase extraction and the preparation of calibration curve

Samples (500 mL) were collected in polypropylene containers, which had been pre-rinsed with ultrapure water. Before sampling of chlorinated water, for chlorine quenching, Sodium thiosulfate was added to chlorinated water samples at a concentration of 20 mg/L [23]. Next, the samples were passed through 1 μm glass-fiber filters and followed by 0.45 μm nylon membrane filters. Na_4_EDTA (375 mg) was added to all samples as a chelating agent to prevent antibiotics from forming complexes with metallic ions [24]. Then filtered samples pH was adjusted to 3 using hydrochloric acid. Oasis HLB cartridges were selected as SPE sorbent, based on preliminary experiments in other studies and prior knowledge from the literature [8, 24–26].

Water samples were extracted with the hydrophilic/lipophilic balanced Oasis HLB (500 mg, 6 cm^3^) for all the matrices in question [8]. The HLB cartridges were conditioned by 10 mL of MeOH followed by 10 mL of HPLC water, which was acidified at pH 2.5 by hydrochloric acid. Both methanol and water were passed through cartridges at a flow rate of 2 mL/min. After that, 500 mL of river water, treated water and ground water samples were passed through the cartridge at a flow rate of 1–2 mL/min. After preconcentration of the samples, 5 mL HPLC water was loaded onto the cartridges at a flow rate of 2 mL/min. The excess water was removed by vacuuming the cartridges for 30 min. Then the analytes were eluted by passing 6 mL methanol through the cartridges at a flow rate of 1 mL/min with the help of gravity in a 10 mL vial. Next, the extract was evaporated to dryness under a gentle stream of nitrogen at 30 °C and finally it was reconstituted with 500 μL of mobile phase (0.1% formic acid in MeOH/HPLC grade water (50:50, *v*/v)). 20 μL of the reconstituted solution was injected into HPLC-ESI-(QqLIT) MS/MS. The linear range was determined by spiking ground water, treated water and river water in the concentration range of 0.5–500 ng/L and fixed amount of the internal standard mixture in the concentration of 80 ng/L. Calibration curves were plotted by the ratio of each antibiotic peak area to peak area of its corresponding internal standard. Regression coefficient (R2) values ranging, varied from 0.95 to 0.99, to a concentration point ranging from 0.5 to 500 ng/L, depending on the target antibiotics. One calibration standards were analyzed repeatedly after every 12 samples in order to check the signal stability.

### High-performance liquid chromatography–tandem mass spectrometry

Chromatographic separations were carried out using an Agilent Ultra High Performance liquid chromatography system (1260 HPLC system), equipped with Micro Vacuum Degasser, Binary pump 600 bar, automated injection system using a Zorbax-eclipse XDB-C18 (100 mm × 4.6 mm, 3.5 μm) column with guard injection 20 μL and oven temperature was 25 °C. The optimum separation conditions were composed of solvent A: HPLC water acidified by adding formic acid at 0.1%, and solvent B: methanol acidified at 0.1% by adding formic acid at a flow rate of 0.8 ml/min. The gradient elution was presented in Table 2.

**Table 2:** The gradient elution of mobile phase

Step	Total Time(min)	Flow Rate(μl/min)	A (%)	B (%)
0	0.00	800	95.0	5.0
1	1.00	800	95.0	5.0
2	5.00	800	12.0	88.0
3	12.00	800	0.0	100.0
4	20.00	800	95.0	5.0

The tandem mass analysis was performed by a 3200 QTRAP hybrid triple quadrupole linear ion trap mass spectrometer (Applied Biosystems, Foster City, CA, USA) equipped with a turbo Ion Spray source. The temperature of the electrospray source was 400 °C. Ion Spry Voltage (IS) was 5500 V. Curtain gas (CUR) was 10 psi and ion source gas pressures (GS1) and (GS2) both of them were 50 psi. MS parameters which dependent to compounds includes (declustering potential (DP), collision energy (CE) and collision cell (CXP)) which were optimized by infusion of individual standard solutions of each compound at concentrations of 2 mg/L. Mass spectrometry analysis (MS) was performed in the positive ion mode (PI) and for each antibiotic [M + H] ^+1^ was selected as precursor ion. It was operated in multiple reaction monitoring mode (MRM). Moreover, for each compound, two MRM transitions were monitored that the most abundant fragment ion was used for quantification and the other one used for identification. A dwell time of 100 ms per ion pair was used for all antibiotics. The resolution on both analyzers were unit. A summary of individual mass parameters is presented in Table 3. The chromatograms for MRM which well-separated by the Zorbax-eclipse XDB-C18 column are presented in Additional file 1: Fig. S3.

**Table 3:** The optimum MRM conditions and retention time (RT) for antibiotics by HPLC/MS/MS

Raw	Antibiotic	Time (msec)	Precursor ion(m/z)	Q3	DP/EP/CEP/CE/CXP	Q3	DP/EP/CEP/CE/CXP	RT(min)
1	Azithromycin-d3	100	753.076 [M + H]+	83.2	71/7.5/64/75/4	158.2	71/7.5/64/51/4	5.096
2	Amoxicillin	100	366.32 [M + H]+	114.3	21/5/36/29/4	134.2	21/5/36/43/4	1.86
3	Cefixime	100	454.699 [M + H]+	126.2	41/6/42/45/4	285.3	41/6/42/23/6	5.22
4	Ceftriaxone	100	555.909 [M + H]+	125.1	31/6/44/77/4	167.2	31/6/44/35/4	4.91
5	Ciprofloxacin	100	332.498 [M + H]+	314.500	46/5.5/36/37/6	231.400	46/5.5/36/47/6	4.96
5	Ciprofloxacin-d8	100	340.578 [M + H]+	322.500	46/7/38/35/6	235.300	46/7/38/51/6	4.9602
6	Erythromycin	100	735.086 [M + H]+	158.200	46/6.5/64/39/4	83.100	46/6.5/64/73/4	5.4607
7	Metronidazole	100	172.103 [M + H]+	128.100	31/4.5/20/19/4	82.1	31/4.5/20/33/4	4.5051
8	Penicillin-G	100	335.480 [M + H]+	91.100	56/7.5/38/65/4	128.200	56/7.5/38/37/4	5.14
9	Cephalexin	100	348.497 [M + H]+	158.1	21/4.5/34/19/4	106.200	21/4.5/34/37/4	4.91
10	Cefuroxime-d3	100	427.888 [M + H]+	324.700	71/6.5/36/23/6	143.200	81/6/38/41/4	9.55
11	Metronidazole-d4	100	176.199 [M + H]+	128.100	31/4.5/16/21/4	82.100	31/4.5/16/33/4	4.45
12	Azithromycin	100	750.048 [M + H]+	158.300	76/7.5/62/51/4	83.100	76/7.5/62/75/4	5.0967

### Statistical method using response surface methodology- central composite design (CCD)

Response surface methodology (RSM) was applied to develop and optimize the processes that are influenced by many variables and their interactions. RSM based on Central Composite design (CCD), which is one of the most common used design was applied to optimize simultaneously three independent variables. Then it was followed by desirability function (DF) to suggest an optimized model [27]. In this purpose, Design_ Expert 7.0.0 (version 7.0.0, Stat-Ease Inc., Minneapolis, MN, USA), was used to generate the experimental matrix and then to evaluate the results [27].

Since, the extraction process is one of the important steps in pharmaceutical analysis including antibiotics and has effects on antibiotics recoveries. Optimization of the extraction process and a careful selection of experimental conditions is crucial. To improve the extraction efficiency, three independent variables pH (X1), the volume of elution solvent (X2) and the amount of Na_4_EDTA (X3) in three levels (low, basal and high) in coded value (−1, 0, +1) and the star points of +1.68 and −1.68 as (+α and –α) were evaluated respectively. Independent variables in maximum and minimum levels were depicted in Table 4. The arrangement of experiments and their responses of all antibiotics are presented in Additional file 1: Table S1.

**Table 4:** Independent variables in maximum and minimum levels and the star point for CCD

Variables	Levels			Star point α = 1.68	
	Low (−1)	Central (0)	High (+1)	−α	+α
(X_1_) pH	3	5.5	8	1.2	9.7
(X_2_) Solvent Volume (mL)	2	4	6	0.63	7.36
(X_3_) Na4 EDTA (mg)	25	50	75	7.955	92.04

In ANOVA tables, a *P*-value less than 0.05 (*P* < 0.05) indicates the statistical significance of effects at 95% confidence interval. According to a second-order polynomial model and to minimize the effect of uncontrolled variables, 20 experiments were designed randomly by Central Composite Design (CCD) and carried out.

## Results and discussion

### Optimization of extraction parameters by CCD

Based on previous knowledge from the literature, three significant variables, pH, the volume of elution solvent and the amount of Na_4_EDTA in the range of 3–8, 2–6 mL and 25–75 mg, were investigated. For this purpose, 100 mL ultrapure water samples were spiked by 80 ng/L of a mixture of antibiotics and then they were extracted by SPE process, which is described in section 2.4, in proposed conditions by CCD.

After doing CCD based-experiments, the quadratic polynomial models were generated from the experimental data (Additional file 1: Table S11). The significance of the models equations was assessed by analysis of variance and checked by F-test which is shown in ANOVA tables (Additional file 1: Tables S2–S10) [28, 29]. The regression coefficients (R^2^), lack of fit, adjusted R^2^ and predicted R^2^ for all target antibiotics were evaluated and presented in Additional file 1: Table S11. The results of two β- lactam antibiotics, amoxicillin and penicillin, were presented and discussed here.

Regarding to Additional file 1: Table S11, the adjusted R^2^ for amoxicillin and penicillin were 0.9696 and 0.9798 respectively, which indicated that more than 90% of the variability within the response data will be explained by model. In general, the higher the R-squared, the better the model fits of data. Adequate Precision for both responses, amoxicillin and penicillin, were 35.550 and 28.906 respectively. The signal to noise ratio (response/deviation) was measured by adequate precision. A ratio greater than four is desirable. The ratios of 35.550 and 28.906 indicate the adequate signal to noise, so the model can be used to navigate the design space.

Reproducibility of the model is measured by the coefficient of variation (C.V). The value of C.V less than 10% indicate that the model is reproducible [29]. the values of C.V for Amoxicillin and Penicillin were 3.69 and 6.99 respectively, that indicates the model is reproducible sufficientlly.

Lack of fit in both ANOVA tables was non-significant with *p*-value of 0.3419 and 0.5758 for amoxicillin and penicillin respectively which Non-significant lack of fit means the model is fit due to the pure error [30, 31]. Lack of fit of the models for all antibiotics were non-significant (Additional file 1: Tables S2–S10). Diagnostic plots including Perturbation plot (A), normal probability plot (B), Internally studentized (C) and plot of residuals versus predicted values (D) were analysed for both responses and depicted in Additional file 1: Figs. S1 and S2.

As can be seen in Additional file 1: Figs. S1B and S2B, in the normal probability plot for both antibiotics, residuals (the points) lie down close to the straight line, which indicate that the residuals follow a regular distribution [29, 31]. Furthermore, the Perturbation plots show the relative significance of factors on extraction recoveries of antibiotics and provides a lay-out view of response surface plots. The steepest slope of curvatures indicate the sensitivity of recovery to pH (Additional file 1: Figs. S1A and S2A) [29–32]. The plot of residual versus predicted value Additional file 1: Figs. S1C and S2C, represent the deviation of actual values versus predicted values. Moreover, They show that residuals were distributed randomly around zero and there wasn’t any outlier. It implies that there isn’t any point with more than three-time of standard deviation from the mean. The actual and predicted Extraction recoveries (ER%) of amoxicillin and penicillin are shown in Additional file 1: Figs. S1D and S2D respectively. Considering that the adjusted R^2^ and predicted R^2^ should be within 0.2 of each other, in this model adjusted R^2^ and predicted R^2^ for ER% of amoxicillin and ER% of Penicillin were (0.9696 and 0.9328) and (0.9798 and 0.9133) respectively. So good agreement between the adjusted R^2^ value and each predicted R^2^ value indicated a proper adjustment between the observed and predicted values [31, 33]*.* Model Equations in terms of coaded factor are depicted in Additional file 1: Table S11.

### The interaction effects of independent variables on the extraction recoveries

**Fig. 1:**
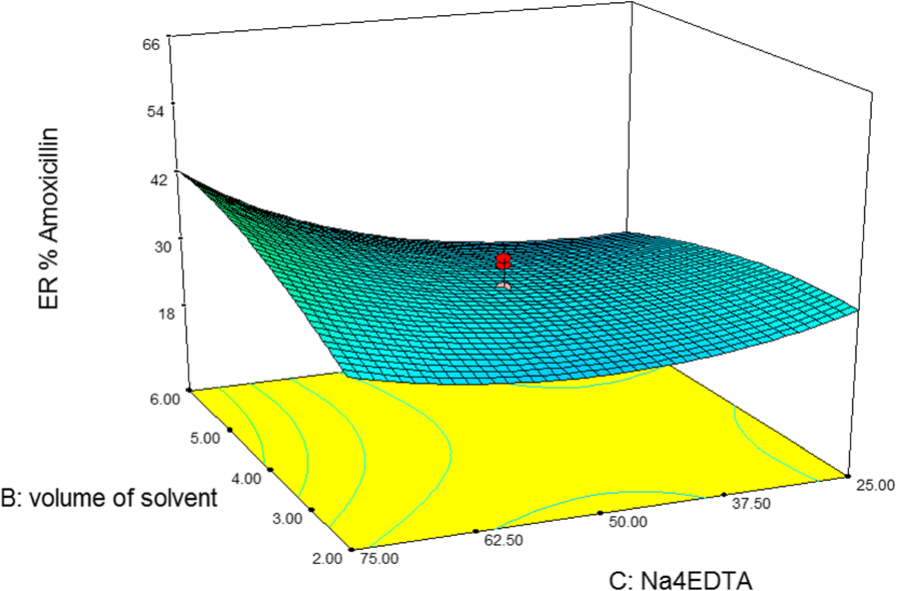
Response surface plot** for ER% Amoxicillin**

**Fig. 2:**
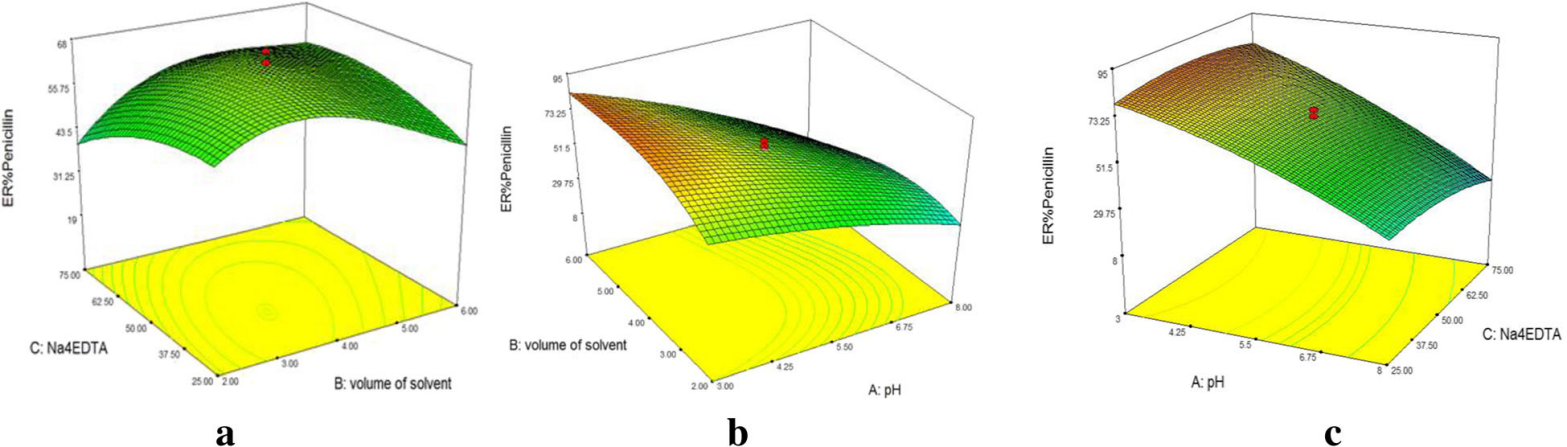
Response surface plot for ER% Penicillin in, **pH value of 5.5, ** (Na4EDTA vs. Volume of solvent) (**a**). Response surface plot for ER% Penicillin **in Na**_**4**_ **EDTA = 50 mg** (pH vs. Volume of solvent) (**b**). Response surface plot for ER% Penicillin **in the volume of solvent = 4 mL** (pH vs. Na4EDTA) (**c**)

Response surface plots depict the extraction efficiencies are a function of two independent variables. The response surface plot for ER% of amoxicillin shows that the increasing in the volume of solvent from 2 to 6 mL led to a marginal increasing of amoxicillin recoveries in both the low and high levels of Na_4_EDTA (Fig. 1). Figure 2a represents that if pH value was kept constant at 5.5, increasing in the amount of Na4EDTA and the volume of extraction solvent led to decreasing of the extraction recovery (ER %) of penicillin up to 64%. While, in pH = 3, with increasing of the amount of Na_4_EDTA and the volume of elution solvent, the highest extraction recovery (ER%) of penicillin was obtained up to 80% (Fig. 2b and c). The response surface plot for ER% of penicillin Fig. 2b, shows that at low levels of pH, the increasing in the volume of the solvent resulted in a rapid enhancement of ER% for Penicillin. Figure 2c shows that at low and high levels of Na_4_EDTA, with increasing in pH levels from 2 to 8, ER% of Penicillin has a rapid decline which can be explained by pKa values of compounds. These surface plots apparently indicated that pH was the main factor which affects the extraction recovery of antibiotics.

### Effect of pH

The pH of the samples plays an important role in the extraction recovery of antibiotics [25]. It can affect the stability and the interaction between the analytes and the material of sorbent. To study the effects of pH on extraction efficiency of antibiotics in water matrices, pka values for all compounds should be investigated. Considering that β-lactams (BLs), macrolides (MLs), fluoroquinolones (FLs), nitro-imidazole antibiotics have acidic or basic functional group, so their ionization is controlled by the pH of the sample solution [21]. Therefore, regarding acidic substances, acidification of water samples 2 unit under pka values of target antibiotics is recommended to obtain their neutral or acidic form of the analytes. It leads to retention of these target antibiotics in Oasis HLB cartridges [8, 21, 34]. In some previous studies, the best recoveries for antibiotics were obtained in strong acidic condition in the range of 2 to 4 [8, 9, 15, 21]. But in some studies pH adjustment has not taken into consideration in extraction process [35], whereas pH adjustment is an important step in extraction efficiency due to different sample pH and charge of each analyte. In this work, the majority of studied analytes were β-lactam antibiotics, including penicillins and cephalosporins, which their analysis was difficult in multi-residues methods due to the chemically unstable β-lactam ring. So that they readily undergo hydrolysis [5, 15, 25, 36, 37]. That’s why, β-lactam antibiotics have been investigated in a few studies [8].

Additional file 1 Table S1 represents the recoveries of β-lactam and cephalosporin antibiotics which were obtained more than those reported for erythromycin, azithromycin and ciprofloxacin. It can be explained by pKa values.

Regarding pKa values of amoxicillin, penicillin, cefixime, cephalexin and ceftriaxone (Table 1), at acidic and natural conditions they are in cationic and zwitterion form, so they were well-retained on the polymeric Oasis HLB column due to their polarity. According previous studies, better recoveries were obtained by Oasis HLB cartridges concern to hydrophilic–lipophilic character of the sorbent [9, 10, 38]. So in present work, higher recoveries for almost all target antibiotics were obtained by acidification of samples prior to extraction process with Oasis HLB cartridges [8, 9, 11, 39–45]. In fact, because of the chemical composition of Oasis HLB, which consists of lipophilic divinylbenzene units and the hydrophilic N-vinylpyrrolidone units, the efficient extraction of organic contaminants in a wide range of pH (from pH 1 to 14) can be obtained. [8] Therefore, the recovery experiments and the analysis of samples were carried out in this conditions.

ANOVA tables show pH was the most significant model terms in extraction recovery of amoxicillin, penicillin, cefixime, cephalexin, ceftriaxone, with *p*-value of <0.0001, < 0.0001, < 0.0001, 0.0002, < 0.0001 respectively (Additional file 1: Tables S2, S3, S5, S7 and S9). Considering to pka values, penicillins and cephalosporins were in a cationic form in acidic condition 2 units under their pk_a_ values. Hence, they were well retained on the polymeric Oasis HLB cartridge [14, 21]*.* In addition, regards to Table 6, the recoveries for amoxicillin, penicillin, cephalexin, ceftriaxone were obtained up to 90%, except for cefixime which were obtained up to 55% in different water matrices. Macrolide antibiotics consist of a basic dimethylamine [−N (CH3)2] group, which is able to take a proton [21] and according to their pk_a_ values, they are basic compounds, so that their retention on reversed phased cartridges was not pH dependent in the range of 3–7 [21, 46]. Therefore, pH was not significant model term in extraction efficiency of azithromycin and erythromycin with *p*-value of 0.3218 and 0.1326 respectively. Regarding to the pk_a_ values of azithromycin (8.74) and erythromycin (8.88) (Table 1) [47], they can be recovered in higher values of pH. Hence, the recoveries of azithromycin and erythromycin in the recovery study were obtained in treated water, ground water and surface water (52, 55 and 47%) and (56, 44 and 42%) respectively (Table 6).

Regarding fluoroquinolones, ciprofloxacin is one of the most widely used fluoroquinolone antibiotics all over the world [48]. They have an amino group (piperazinyl) in the heterocyclic ring and have two dissociation constants [8]. The reported pKa values of ciprofloxacin were 5.76 (strangest acidic) and 8.68 (strangest basic) [49, 50]. They are in their zwitterion form in neutral condition and in cationic form in acidic condition so they are well retained on the polymeric Oasis HLB column [21]. The recoveries of ciprofloxacin in treated water, ground water and surface water were 75, 88 and 87% respectively (Table 6). For compounds with relatively high pKa values such as metronidazole, the improvement in recovery is not significant under sample acidification [9]. Moreover, the result of ANOVA test for metronidazole shows that pH model term was not statistically significant (*p*-value = 0.1134) in extraction efficiency of metronidazole (Additional file 1: Table S8).

### Effect of eluent volume

In order to desorb the adsorbates as fully as possible in SPE columns and to maximize the sensitivity of the analysis, to choose appropriate eluent is essential. According to the fact that SPE extraction is based on the interaction between adsorbate and adsorbent, extraction efficiency is highly dependent on the polarity of the eluent [9]. Acetonitrile and methanol were the most frequently used eluents in the extraction recovery of antibiotics from HLB cartridges [8, 39]. Although methanol can cause the potential for analyte degradation [44, 51] it can provide better chromatographic peaks. If chromatographic run is fast enough, degradation effects can be negligible [44, 51]. So methanol was used as elution solvent in this work. The volume of elution solvent was the statistically significant model term in extraction efficiency of four antibiotics included cefixime, metronidazole, ceftriaxone and azithromycin with *p*-value of <0.0001, 0.0113, 0.0002 and 0.0094 respectively. It is noteworthy that, large volume of elution solvent more than 6 ml takes more time in evaporation step in SPE process, degradation of β- lactams is quite likely in the presence of methanol under the gentle nitrogen stream due to instability of β- lactam ring. To avoid analytes degradation and shorten the time of evaporation, the extracts were evaporated under a gentle nitrogen stream in 35 °C in a water bath which shortened the time of evaporation up to 1.5 h.

### Effect of Na_4_EDTA

Due to the fact that the antibiotics can potentially bind residual metals which are present in the sample matrix and glassware, the extraction recoveries decrease. By adding chelating agent, including Na_4_EDTA, metal ions are bounded to the chelating agent and make the extraction efficiency increase [10, 52, 53]. The effect of Na_4_EDTA ranged from 25 to 75 mg/L on extraction efficiency was evaluated. Regarding to ANOVA tables (Additional file 1: Tables S2–S10), the value of Na_4_EDTA was not a significant model term in extraction efficiency except for amoxicillin with p-value of 0.0007. On the other hand, simultaneous increase of Na4EDTA and the volume of elution solvent lead to decreasing of extraction efficiency (Fig. 4a). Fluoroquinolones and macrolides have a high tendency to complex with metal ions which resulting in lower extraction recoveries. So, to achieve higher extraction efficiency, the addition of strong enough chelating agent is crucial [8].

### Optimization of CCD by desirability factor for extraction procedure

The optimization profile in Expert Design 7.0.0 software was used to optimize the extraction process. The desirability is measured by specifying the desirability factor (DF) for each dependent variable or responses. It can take values from 0 (undesirable) to 1 (very desirable). It would be maximized if the selected criteria were optimized efficiently. The criteria for optimization were pH, Na_4_EDTA and the volume of extraction solvent. Desirability function (D) is expressed by the equation follows:

1 $$ \mathrm{D}={\left[{{\mathrm{d}}_1}^{\mathrm{p}2}{{\mathrm{xd}}_2}^{\mathrm{p}2}{{\mathrm{xd}}_3}^{\mathrm{p}2}\mathrm{x}\dots \dots {{\mathrm{d}}_{\mathrm{n}}}^{\mathrm{p}\mathrm{n}}\mathrm{x}\right]}^{1/\mathrm{n}} $$

Where p^i^ is the weight of the response, n the number of responses and d_i_ is the individual desirability function of each response [29, 31, 32]. The criteria can be weighted from 0.1 to 10 which lower than 1 gives less importance to the goal and greater than 1 gives more importance. Five suggested models with desirability up to 0.84% were presented in Additional file 1: Table S12. In method validation procedure, the validity of experiments in optimum operating conditions was investigated, and the results were close to which obtained from the optimized model using a CCD.

### Method validation

Quantification was based on linear regression calibration curves using the internal standard approach to minimize or correct the matrix effects. The method validation was assessed in terms of linearity; accuracy and precision, instrumental detection limit (IDL), method detection limit (MDL), method quantification limit (MQL) and repeatability which the results were presented in Tables 5 and 6. Accuracy was assessed by further doing experiments using spiking natural water samples. Recoveries were determined by spiking treated water, ground water and river water, in triplicate, at a concentration of 100 ng/L of a mixture of antibiotics and extracted under the optimum conditions, which the recoveries were presented in Table 6. Two standard mixtures were spiked, the first one contained penicillins and cephalosporins in HPLC grade water and the other one contained azithromycin, erythromycin and metronidazole in methanol [8]. Recoveries obtained for all antibiotics ranged from 50 to 110% except metronidazole which was obtained in treated, ground and river water 43%, 45% and 41% respectively. Furthermore, erythromycin in treated water, azithromycin and erythromycin in river water samples showed low recoveries (44%, 47% and 42% respectively). The low recoveries of some macrolide antibiotics and metronidazole can be described by instability in acidic condition through SPE process. Regarding some cephalosporins, low recoveries in all water matrices is due to their chemical structure and undergoing the hydrolysis [21]. In present work, the reported MDLs and MQLs for penicillins and cephalosporins are comparable to those were obtained in previous literatures [8, 14, 54].

**Table 5:** Calibration equation, instrumental detection limits (IDLs), linearity and repeatability (run-to-run analysis) determined for target antibiotics

No.	Antibiotic	Calibration equation	Regression coefficient	IDL (ng.L^−1^)^a^ (injected)	Repeatability %RSD (*n* = 5)
1	Metronidazole	y = 0. 172X + 0.324	0.992	1	13.4
2	Ceftriaxone	y = 5.32 e-4X- 4.7e-4	0.984	2.5	11.6
3	Penicillin	y = 0. 0067X- 0.058	0.991	2.5	8.1
4	Amoxicillin	y = 0. 028 X- 6.8e-4	0.956	1	5.3
5	Azithromycin	y = 0. 052X- 0.32	0.974	1	7.8
6	Cephalexin	y = 0. 003X- 0.024	0.991	2.5	5.1
7	Ciprofloxacin	y = 0. 013X- 0.042	0.993	2.5	8.4
8	Erythromycin	y = 0. 015X + 0.36	0.987	1	6.7
9	Cefixime	y = 5. 8e-4X + 0.02	0.990	2.5	6.3

^a^ *IDL* Instrumental Detection Limit

**Table 6:** Method validation parameters including, recoveries obtained for target antibiotics, method detection limits (MDLs), and method quantitation limits (MQLs) in treated water, ground water and river water matrices

No.	Antibiotic	Treated water				Ground water				River water			
		Mean recovery (%)	RSD (%) (*n* = 3)	MDL^a^ ng/L (Spiked)	MQL^b^ ng/L (Spiked)	Mean Recovery (%)	RSD (%) (n = 3)	MDL^a^ ng/L (Spiked)	MQL^b^ ng/L (Spiked)	Mean recovery (%)	RSD (%) (n = 3)	MDL^a^ ng/L (Spiked)	MQL^b^ ng/L (Spiked)
1	Metronidazole	43	7.3	5	10	45	6.4	5	10	41	8.1	10	25
2	Ceftriaxone	108	3.8	10	25	113	9.8	10	25	107	8.6	10	25
3	Penicillin	107	5.6	5	10	101	4.6	5	10	90	3.6	5	10
4	Amoxicillin	96	6.1	2	5	109	7.4	2	5	95	6	2.5	5
5	Azithromycin	52	7.6	1	2.5	55	7.1	0.5	2	47	4.5	0.8	2.5
6	Cephalexin	117	4.9	5	25	120	5.5	5	25	111	7.3	5	25
7	Ciprofloxacin	75	7.3	2.5	5	88	6.1	2	5	85	6.5	2.5	5
8	Erythromycin	56	8.6	1	5	44	3.7	0.8	2	42	8.9	2.5	5
9	Cefixime	55	9.3	10	25	57	7.4	25	50	53	8.5	25	50

^a^ Method Detection Limit

^b^ Method Quantification Limit

RSD values were determined for all antibiotics in natural water samples, which were less than 10%, which was a good signal of precision. RSD values were in the range of 3.8–9.3% for treated water, (3.7–9.8%) for ground water and (3.6–8.9%) for river water. Regarding standard deviation values calculated for each antibiotic (RSD, *n* = 3), repeatability of the proposed method is high. The method detection limit (MDL) and method quantification limit (MQL) were calculated with a signal to noise of 3 and 10 respectively. But finally we checked it with our findings in practice. According Table 6, MDL values varied in the range of 1–10 ng/L in treated water, 0.8–10 ng/L in the ground water and 0.8–25 ng/L in river water. MQL values for treated water were ranging from 2.5–25 ng/L, ground water 2–50 ng/L and river water 2.5–50 ng/L. Repeatability was assessed by five consecutive injection of a 500 ng/L calibration curve standard. The RSD values were achieved below 10% with exception for metronidazole and ceftriaxone.

### Application to natural water matrices

The developed method was successfully applied to treated water, ground water and river water samples which were collected from Tehran water resources, including water wells and drinking water treatment plants and two rivers passing through residential zones in Tehran during July to September in 2016. Sixty treated water, 39 ground water and 12 river water samples were collected and analyzed. The results are summarized in Table 7. None of the antibiotics were detected in treated water and ground water samples but seven out of nine antibiotics were detected in Kan River water and Firozabad Ditch. Ceftriaxone and metronidazole were not detected in any of the samples. The concentrations of antibiotics varied from 11.76 ng/L for ciprofloxacin in Kan River samples to 404.96 ng/L for azithromycin in Firozabad Ditch.

**Table 7:** Detected antibiotics in ng/L, in Treated water, in Ground water and surface water including, Kan River and Firozabad Ditch

No.	Antibiotic	Treated water (*n* = 30)	Ground water (*n* = 39)	Kan River water (*n* = 6)	Firozabad Ditch (n = 6)
1	Metronidazole	N.D^a^	N.D	N.D	N.D
2	Ceftriaxone	N.D	N.D	N.D	<MQL^b^
3	Penicillin	N.D	N.D	50.94 ± 17.68	21.05 ± 12.53
4	Amoxicillin	N.D	N.D	18.00 ± 11.03	31.14 ± 11.67
5	Azithromycin	N.D	N.D	52.66 ± 0.14	404.96 ± 175.46
6	Cephalexin	N.D	N.D	96.03 ± 87.29	94.51 ± 55.35
7	Ciprofloxacin	N.D	N.D	11.76 ± 7.42	308.65 ± 253.58
8	Erythromycin	N.D	N.D	16.61 ± 1.03	28.055 ± 11.05
9	Cefixime	N.D	N.D	71.89 ± 73.62	79.09 ± 49.47

^a^ Not detected

^b^ Below method quantification limit

The highest concentration of antibiotics was discovered in Firozabad Ditch which is receiving the effluent of Ekbatan wastewater treatment plant located in Ekbatan area in west of Tehran. Furthermore, cefixime and cephalexin were detected at the highest median concentration of 71.91 and 93.19 ng/L in Kan River samples respectively. While azithromycin and ciprofloxacin were detected at the highest median concentration of 439.35 ng/L and 212.83 ng/L respectively.

The discovered antibiotics in the Kan River were attributed to the discharges of untreated wastewaters from the unauthorized constructions along the river and releasing the untreated wastewater directly into the river. It is not surprising that azithromycin and ciprofloxacin were detected at the highest median concentrations. The presence of antibiotics belonging to macrolides and fluoroquinolones (erythromycin, azithromycin and ciprofloxacin) had also been reported in surface water in previous works [40, 55–57]. Although many researchers detected different classes of antibiotics in wastewater treatment plant influent and effluent as well as hospital wastewater [8, 58, 59], a few works reported our target antibiotics in surface water. Gros et al. (2006) found erythromycin and azithromycin in surface water in the range of <MDL – 30 ng/L and <MDL – 20 ng/L respectively [11], though at lower levels than those reported herein. Hao et al., (2011) detected azithromycin and erythromycin in the highest concentration of 90.80 and 2246 ng/L in the Red River delta of northern Vietnam [60]. Christian et al., (2003) detected azithromycin, erythromycin and ciprofloxacin in the range of 2–3 ng/L, 4–190 ng/L and 9 ng/L in the aquatic environment of the Land of North Rhine-Westphalia in Germany [61]. The presence of erythromycin was quantified with 0.62 μg/l in the small river Lutter in Bielefeld, Germany [62]. The presence of erythromycin, azithromycin and ciprofloxacin were detected in the range of 50.38–174.73 ng/L, 14.73–71.76 ng/L and 8.32–28.02 ng/L respectively in Llobreg River basin, NE Spain [26]. Regarding to previous literatures, azithromycin and erythromycin and ciprofloxacin are frequently detected antibiotics in surface water [26, 61, 63]. Penicillin and Amoxicillin were reported in wastewater samples and surface water frequently but studies including cephalosporins are still infrequent. In present work, cephalosporin antibiotics including cefixime and cephalexin were detected in the range of <MQL- 136.31 ng/L and <MQL – 177.60 ng/L in both investigated river waters, respectively. That high concentrations of antibiotics in Kan River and Firozabad Ditch were found in dry season and the other reason is probable due to low rainfall and untreated wastewater discharges.

**Fig. 3:**
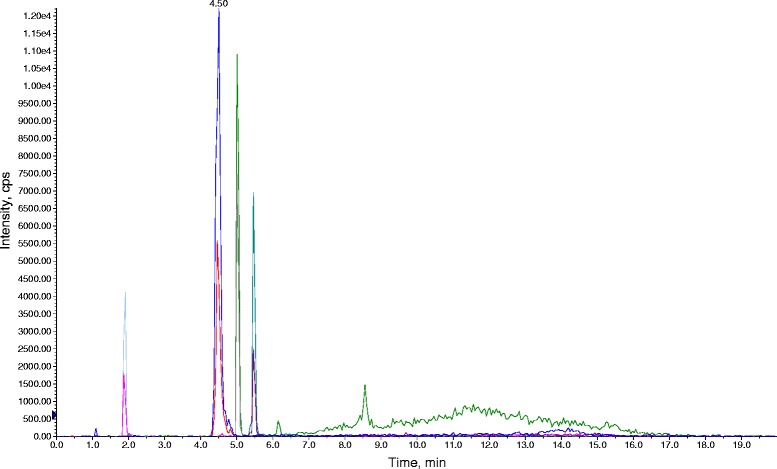
Total ion chromatograms (TIC) of a 50 ng/mL standard mixture of target antibiotics

**Fig. 4:**
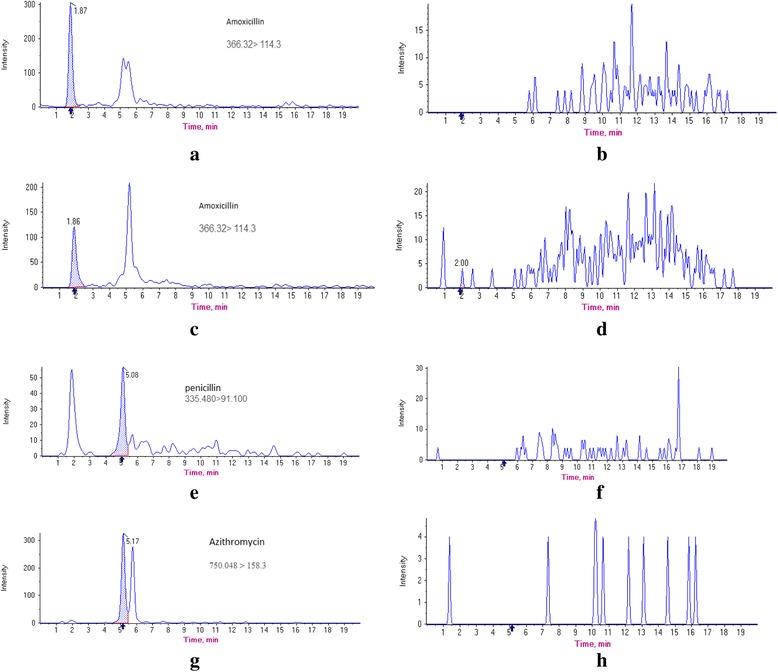
Chromatograms for β- lactam antibiotics and one macrolide antibiotic found positive in two river water samples: **a** Amoxicillin at 31 ng/L in Firozabad stream water sample; **b** chromatogram of corresponding blank of amoxicillin (RT = 1.87 min), and **c** 18 ng/L in Kan River sample, **d** chromatogram of corresponding blank of amoxicillin (RT = 1.87 min), **e** penicillin at 51 ng/L in Kan River sample; **f** chromatogram of corresponding blank of penicillin (RT = 5.08 min); **g** Azithromycin at 404 ng/L in Firozabad stream water; **h** chromatogram of corresponding blank of Azithromycin (RT = 5.17 min)

In Fig. 3, the total ion current (TIC) chromatograms from a standard mixture at a concentration of 50 ng/mL is depicted and the chromatograms for real samples containing some of the compounds analyzed are displayed in Fig. 4.

## Conclusion

An optimized SPE-HPLC-MS/MS method developed to analyze multi-residues of different classes of antibiotics, including β-lactam (amoxicillin and penicillin G), cephalosporin (ceftriaxone, Cefixime, and cephalexin), macrolides (azithromycin and erythromycin), fluoroquinolone (ciprofloxacin) and Nitro-imidazole (metronidazole) in groundwater and treated water as well as surface water. PH, the amount of Na_4_EDTA and the volume of elution solvent were simultaneously optimized by applying response surface methodology – central composite design to explore the relationship between variables and responses (extraction recoveries of antibiotics) in the extraction process. D-optimal function was applied to suggest the predicted models and report desirability factors (DF) for suggested models. After doing CCD- based experiments, the results showed that the acidification of water samples to pH 3 improved the retention of β-lactam and cephalosporin antibiotics on HLB cartridges for all water matrices which the results were in accordance with the pka value of each compound. The optimum volume of elution solvent and the optimum amount of Na_4_EDTA was obtained 6 ml and 75 mg in (100 ml of water sample) respectively. Analysis of variance was carried out for nine antibiotics and pH was the most significant model term in model equations for all antibiotics expect azithromycin, erythromycin and metronidazole which were in agreement with their pKa values. The volume of elution solvent was significant model terms for cefixime, ceftriaxone and metronidazole model equations. While, the amount of Na_4_EDTA was the significant model term in the amoxicillin model equation. The developed method resulted in method detection limit in the range of low ng/L for all water matrices and provided a reliable analytical performance within multi-residues method for β-lactam antibiotics including penicillin and cephalosporins. The developed method was applied to treated, ground and river water samples. Seven out of nine antibiotics were detected in Kan River and Firozabad Ditch water samples, although none of them were detected in treated water and ground water samples.

## Additional file

Additional file 1: It was provided in a format of DOC (Microsoft Word) including Central composite arrangement and responses in Table S.1 and ANOVA Tables S.2-10, The quadratic polynomial models and statistical parameters in Table S.11, Five proposed models for analysis of investigated antibiotics by multiresidues method in Table S.12, d iagnostic plots for ER% of amoxicillin in Fig.S.1, diagnostic plots for ER% of penicillin in Fig.S.2 and the chromatograms for MRM by the Zorbax-eclipse XDB-C18 column in Fig.S.3 were depicted. (DOCX 6167 kb)

## Abbreviations

CCD: Central Composite Design
CE: Collision energy
CV: Coefficient variation
CXP: Cell exit potential
DF: Desirability factor
DP: Declustering potential
EDTA: Ethylenediaminetetraacetic Acid
ESI: Electrospray ionization
HLB: Hydrophilic-Lipophilic Balance
HPLC: High Performance liquid chromatography
IS: Internal standard
LC-MS/MS: Liquid Chromatography Tandem Mass Spectrometry
LOD: Limit of Detection
LOQ: Limit of Quantification
MDL: Method detection limit
MQL: Method quantification limit
RSM: Response Surface Methodology
