# Supplementary material for: An optimized SPE-LC-MS/MS method for antibiotics residue analysis in ground, surface and treated water samples by response surface methodology- central composite design
Source: J Environ Health Sci Eng. 2017 Oct 17;15:21. doi: 10.1186/s40201-017-0282-2 (PMC5646162; doi:10.1186/s40201-017-0282-2)
Supplement: Additional file 1: — It was provided in a format of DOC (Microsoft Word) including Central composite arrangement and responses in Table S.1 and ANOVA Tables S.2-10, The quadratic polynomial models and statistical parameters in Table S.11, Five proposed models for analysis of investigated antibiotics by multiresidues method in Table S.12, d iagnostic plots for ER% of amoxicillin in Fig.S.1, diagnostic plots for ER% of penicillin in Fig.S.2 and the chromatograms for MRM by the Zorbax-eclipse XDB-C18 column in Fig.S.3 were depicted. (DOCX 6167 kb) [file 40201_2017_282_MOESM1_ESM.docx]

**Supplementary Materials**

**An optimized SPE-LC-MS/MS method for antibiotics residue analysis in ground, surface and treated water samples by Response Surface Methodology- Central Composite Design**

Roya Mirzaei^1^, Masoud Yunesian^2, 3^, Simin Nasseri^1, 2^, Mitra Gholami^4^, Esfandiyar Jalilzadeh^5^, Shahram Shoeibi^6^, Hooshang Shafieyan Bidshahi^6^, Alireza Mesdaghinia^1, 2^

*^1.^ Center for Water Quality Research (CWQR), Institute for Environmental Research (IER), Tehran University of Medical Sciences, 8th floor, Gol Building, North Karegar St., Enghelab Sq, Tehran, Iran.*

*^2.^ Department of Environmental Health Engineering, School of Public Health, Tehran University of Medical Sciences, Tehran, Iran.*

*^3.^ Center for Air Pollution Research (CAPR), Institute for Environmental Research (IER), Tehran University of Medical Sciences, Tehran, Iran.*

*^4.^ Research center for environmental health technology, Department of Environmental Health Engineering, School of Public Health, Iran University of Medical Sciences, Tehran, Iran.*

*^5.^ Water and Wastewater Company, Department of Water and Wastewater Quality Control Laboratory, Tehran, Iran.*

*^6.^ Food and Drug Reference Control Laboratories Center, Food and Drug Organization, Ministry of Health & Medical Education, Tehran, Iran.*

Correspondence to:

Alireza Mesdaghinia, Center for Water Quality Research (CWQR), Institute for Environmental Research (IER), Tehran University of Medical Sciences, 8th floor, Gol Building, North Karegar St., Enghelab Sq., Tehran, Iran, Tel.:

+98 2188978399, Fax: +982188978398, E-mail: mesdaghinia@sina.tums.ac.ir.

**Table S.1**

Central composite arrangement and responses at concentration of 100 µg/L of antibiotics mixture in ultrapure water (100 mL sample size)

| Run | A:pH | B:volume of solvent (mL) | C:Na4EDTA | ER (%)  Amoxicillin | ER (%) Penicillin | ER (%) azithromycin | ER (%) cefixime | ER (%) Cephalexin | ER (%) Ciprofloxacin | ER (%) Erythromycin | ER (%)  Metronidazole | ER (%) Ceftriaxone |
| --- | --- | --- | --- | --- | --- | --- | --- | --- | --- | --- | --- | --- |
| 1 | 5.5 | 0.64 | 50 | 18 | 19 | 6 | 5 | 17 | 1 | 4 | 3 | 3 |
| 2 | 5.5 | 4 | 50 | 26 | 65 | 18 | 68 | 40 | 2 | 20 | 16 | 52 |
| 3 | 8 | 2 | 75 | 13 | 17 | 68 | 25 | 19 | 37 | 72 | 68 | 20 |
| 4 | 3 | 2 | 75 | 75 | 47 | 65 | 30 | 74 | 45 | 69 | 67 | 29 |
| 5 | 1.3 | 4 | 50 | 93 | 95 | 95 | 98 | 98 | 25 | 99 | 95 | 95 |
| 6 | 8 | 6 | 25 | 10 | 20 | 83 | 24 | 23 | 72 | 88 | 83 | 25 |
| 7 | 5.5 | 4 | 7.96 | 35 | 55 | 15 | 57 | 50 | 5 | 17 | 15 | 56 |
| 8 | 8 | 6 | 75 | 17 | 10 | 51 | 37 | 19 | 44 | 55 | 51 | 35 |
| 9 | 5.5 | 4 | 50 | 26 | 65 | 50 | 72 | 39 | 7 | 55 | 37 | 69 |
| 10 | 3 | 2 | 25 | 76 | 70 | 60 | 58 | 66 | 2 | 62 | 60 | 57 |
| 11 | 5.5 | 7.36 | 50 | 26 | 55 | 51 | 79 | 44 | 7 | 52 | 52 | 75 |
| 12 | 5.5 | 4 | 92.04 | 66 | 49 | 19 | 51 | 45 | 1 | 20 | 17 | 49 |
| 13 | 5.5 | 4 | 50 | 26 | 65 | 19 | 53 | 65 | 2 | 21 | 36 | 51 |
| 14 | 5.5 | 4 | 50 | 32 | 68 | 48 | 67 | 64 | 12 | 50 | 45 | 65 |
| 15 | 5.5 | 4 | 50 | 33 | 62 | 39 | 70 | 59 | 10 | 42 | 44 | 72 |
| 16 | 5.5 | 4 | 50 | 28 | 57 | 40 | 59 | 58 | 8 | 42 | 43 | 60 |
| 17 | 3 | 6 | 25 | 48 | 69 | 90 | 88 | 65 | 12 | 96 | 95 | 79 |
| 18 | 8 | 2 | 25 | 15 | 36 | 41 | 26 | 25 | 33 | 42 | 41 | 23 |
| 19 | 3 | 6 | 75 | 88 | 88 | 96 | 75 | 89 | 19 | 99 | 92 | 78 |
| 20 | 9.7 | 4 | 50 | 8 | 8 | 76 | 35 | 12 | 20 | 80 | 79 | 37 |

**Table S.2**

ANOVA Table for **amoxicillin**:

| Response 1 ***ER % amoxicillin*** | | | | | | |
| --- | --- | --- | --- | --- | --- | --- |
| Transform: Natural log , Constant: 0 | | | | | | |
| ANOVA for Response Surface Reduced Quadratic Model | | | | | | |
| Analysis of variance table [Partial sum of squares - Type III] | | | | | | |
| Source | Sum of  Squares | df | Mean  Square | F  Value | p-value  Prob > F |  |
| Model | 9.62 | 6 | 1.60 | 101.86 | < 0.0001 | significant |
| A-pH | 8.40 | 1 | 8.40 | 533.94 | < 0.0001 |  |
| B-volume of elution solvent | 2.414E-003 | 1 | 2.414E-003 | 0.15 | 0.7017 |  |
| C-Na4EDTA | 0.31 | 1 | 0.31 | 19.50 | 0.0007 |  |
| BC | 0.21 | 1 | 0.21 | 13.28 | 0.0030 |  |
| B^2 | 0.13 | 1 | 0.13 | 8.10 | 0.0138 |  |
| C^2 | 0.52 | 1 | 0.52 | 32.90 | < 0.0001 |  |
| Residual | 0.20 | 13 | 0.016 |  |  |  |
| Lack of Fit | 0.14 | 8 | 0.018 | 1.49 | 0.3419 | not significant |
| Pure Error | 0.060 | 5 | 0.012 |  |  |  |
| Cor Total | 9.83 | 19 |  |  |  |  |

**Table S.3**

ANOVA Table for **Penicillin**

| Response 2 ***ER% Penicillin*** | | | | | | |
| --- | --- | --- | --- | --- | --- | --- |
| ANOVA for Response Surface Reduced Cubic Model | | | | | | |
| Analysis of variance table [Partial sum of squares - Type III] | | | | | | |
| Source | Sum of  Squares | df | Mean  Square | F  Value | p-value  Prob > F |  |
| Model | 11883.04 | 12 | 990.25 | 77.92 | < 0.0001 | significant |
| A-pH | 8331.50 | 1 | 8331.50 | 655.57 | < 0.0001 |  |
| B- volume of elution solvent | 18.71 | 1 | 18.71 | 1.47 | 0.2643 |  |
| C-Na4EDTA | 18.00 | 1 | 18.00 | 1.42 | 0.2728 |  |
| AB | 496.12 | 1 | 496.12 | 39.04 | 0.0004 |  |
| AC | 78.13 | 1 | 78.13 | 6.15 | 0.0422 |  |
| BC | 325.13 | 1 | 325.13 | 25.58 | 0.0015 |  |
| A^2 | 298.62 | 1 | 298.62 | 23.50 | 0.0019 |  |
| B^2 | 1349.99 | 1 | 1349.99 | 106.23 | < 0.0001 |  |
| C^2 | 275.88 | 1 | 275.88 | 21.71 | 0.0023 |  |
| ABC | 136.13 | 1 | 136.13 | 10.71 | 0.0136 |  |
| A^2C | 18.16 | 1 | 18.16 | 1.43 | 0.2708 |  |
| B^3 | 243.82 | 1 | 243.82 | 19.19 | 0.0032 |  |
| Residual | 88.96 | 7 | 12.71 |  |  |  |
| Lack of Fit | 17.63 | 2 | 8.81 | 0.62 | 0.5758 | not significant |
| Pure Error | 71.33 | 5 | 14.27 |  |  |  |
| Cor Total | 11972.00 | 19 |  |  |  |  |

**Table S.4**

ANOVA Table for **Erythromycin**

| ANOVA for Response Surface Reduced Quadratic Model ***ER% Erythromycin*** | | | | | | |
| --- | --- | --- | --- | --- | --- | --- |
| Analysis of variance table [Partial sum of squares - Type III] | | | | | | |
| Source | Sum of  Squares | df | Mean  Square | F  Value | p-value  Prob > F |  |
| Model | 11228.06 | 3 | 3742.69 | 12.60 | 0.0002 | significant |
| A-pH | 746.27 | 1 | 746.27 | 2.51 | 0.1326 |  |
| B- volume of elution solvent | 2209.93 | 1 | 2209.93 | 7.44 | 0.0149 |  |
| A^2 | 8271.85 | 1 | 8271.85 | 27.84 | < 0.0001 |  |
| Residual | 4753.69 | 16 | 297.11 |  |  |  |
| Lack of Fit | 3676.36 | 11 | 334.21 | 1.55 | 0.3287 | not significant |
| Pure Error | 1077.33 | 5 | 215.47 |  |  |  |
| Cor Total | 15981.75 | 19 |  |  |  |  |

**Table S.5**

ANOVA Table for **Cefixime**

| ANOVA for Response Surface Reduced Quadratic Model  ***ER% Cefixime*** | | | | | | |
| --- | --- | --- | --- | --- | --- | --- |
| Analysis of variance table [Partial sum of squares - Type III] | | | | | | |
| Source | Sum of  Squares | df | Mean  Square | F  Value | p-value  Prob > F |  |
| Model | 10266.12 | 7 | 1466.59 | 16.79 | < 0.0001 | significant |
| A-pH | 4393.54 | 1 | 4393.54 | 50.29 | < 0.0001 |  |
| B- volume of elution solvent | 3212.34 | 1 | 3212.34 | 36.77 | < 0.0001 |  |
| C-Na4EDTA | 111.89 | 1 | 111.89 | 1.28 | 0.2799 |  |
| AB | 528.12 | 1 | 528.12 | 6.04 | 0.0301 |  |
| AC | 351.13 | 1 | 351.13 | 4.02 | 0.0681 |  |
| B^2 | 1363.64 | 1 | 1363.64 | 15.61 | 0.0019 |  |
| C^2 | 430.21 | 1 | 430.21 | 4.92 | 0.0465 |  |
| Residual | 1048.43 | 12 | 87.37 |  |  |  |
| Lack of Fit | 781.60 | 7 | 111.66 | 2.09 | 0.2168 | not significant |
| Pure Error | 266.83 | 5 | 53.37 |  |  |  |
| Cor Total | 11314.55 | 19 |  |  |  |  |

**Table S.6**

ANOVA Table for **Cephalexin**

| ANOVA for Response Surface Reduced Cubic Model ***ER% Cephalexin*** | | | | | | |
| --- | --- | --- | --- | --- | --- | --- |
| Analysis of variance table [Partial sum of squares - Type III] | | | | | | |
|  | Sum of |  | Mean | F | p-value |  |
| Source | Squares | df | Square | Value | Prob > F |  |
| Model | 10543.12 | 9 | 1171.46 | 12.28 | 0.0003 | significant |
| A-pH | 9105.38 | 1 | 9105.38 | 95.46 | < 0.0001 |  |
| B-volume of solvent | 241.32 | 1 | 241.32 | 2.53 | 0.1428 |  |
| C-Na4EDTA | 13.53 | 1 | 13.53 | 0.14 | 0.7144 |  |
| AB | 32.00 | 1 | 32.00 | 0.34 | 0.5753 |  |
| AC | 220.50 | 1 | 220.50 | 2.31 | 0.1594 |  |
| BC | 40.50 | 1 | 40.50 | 0.42 | 0.5293 |  |
| A^2 | 17.08 | 1 | 17.08 | 0.18 | 0.6811 |  |
| B^2 | 826.56 | 1 | 826.56 | 8.67 | 0.0147 |  |
| C^2 | 35.20 | 1 | 35.20 | 0.37 | 0.5571 |  |
| Residual | 953.83 | 10 | 95.38 |  |  |  |
| Lack of Fit | 271.00 | 5 | 54.20 | 0.40 | 0.8333 | not significant |
| Pure Error | 682.83 | 5 | 136.57 |  |  |  |
| Cor Total | 11496.95 | 19 |  |  |  |  |

**Table S.7**

ANOVA Table for **Ciprofloxacin**

| ANOVA for Response Surface Reduced Quadratic Model ***ER% Ciprofloxacin*** | | | | | | |
| --- | --- | --- | --- | --- | --- | --- |
| Analysis of variance table [Partial sum of squares - Type III] | | | | | | |
| Source | Sum of  Squares | df | Mean  Square | F  Value | p-value  Prob > F |  |
| Model | 11.38 | 2 | 5.69 | 4.60 | 0.0252 | significant |
| A-pH | 1.73 | 1 | 1.73 | 1.40 | 0.2528 |  |
| A^2 | 9.65 | 1 | 9.65 | 7.81 | 0.0125 |  |
| Residual | 21.02 | 17 | 1.24 |  |  |  |
| Lack of Fit | 17.80 | 12 | 1.48 | 2.31 | 0.1827 | not significant |
| Pure Error | 3.21 | 5 | 0.64 |  |  |  |
| Cor Total | 32.40 | 19 |  |  |  |  |

**Table S.8**

ANOVA Table for **Metronidazole**

| ANOVA for Response Surface Quadratic Model ***ER% Metronidazole*** | | | | | | |
| --- | --- | --- | --- | --- | --- | --- |
| Analysis of variance table [Partial sum of squares - Type III] | | | | | | |
| Source | Sum of  Squares | df | Mean  Square | F  Value | p-value  Prob > F |  |
|  | Sum of |  | Mean | F | p-value |  |
| Source | Squares | df | Square | Value | Prob > F |  |
| Model | 10616.48 | 3 | 3538.83 | 14.14 | < 0.0001 | significant |
| A-pH | 701.93 | 1 | 701.93 | 2.80 | 0.1134 |  |
| B-volume of solvent | 2052.11 | 1 | 2052.11 | 8.20 | 0.0113 |  |
| A^2 | 7862.44 | 1 | 7862.44 | 31.41 | < 0.0001 |  |
| Residual | 4004.47 | 16 | 250.28 |  |  |  |
| Lack of Fit | 3413.63 | 11 | 310.33 | 2.63 | 0.1482 | not significant |
| Pure Error | 590.83 | 5 | 118.17 |  |  |  |
| Cor Total | 14620.95 | 19 |  |  |  |  |

**Table S.9**

ANOVA Table for **Ceftriaxone**

| ANOVA for Response Surface Quadratic Model ***ER% Ceftriaxone*** | | | | | | |
| --- | --- | --- | --- | --- | --- | --- |
| Analysis of variance table [Partial sum of squares - Type III] | | | | | | |
| Source | Sum of  Squares | df | Mean  Square | F  Value | p-value  Prob > F |  |
| Model | 9780.53 | 9 | 1086.73 | 10.78 | 0.0005 | significant |
| A-pH | 4131.78 | 1 | 4131.78 | 40.97 | < 0.0001 |  |
| B-volume of solvent | 3201.19 | 1 | 3201.19 | 31.74 | 0.0002 |  |
| C-Na4EDTA | 83.52 | 1 | 83.52 | 0.83 | 0.3842 |  |
| AB | 364.50 | 1 | 364.50 | 3.61 | 0.0865 |  |
| AC | 162.00 | 1 | 162.00 | 1.61 | 0.2337 |  |
| BC | 200.00 | 1 | 200.00 | 1.98 | 0.1894 |  |
| A^2 | 0.87 | 1 | 0.87 | 8.632E-003 | 0.9278 |  |
| B^2 | 1381.72 | 1 | 1381.72 | 13.70 | 0.0041 |  |
| C^2 | 362.99 | 1 | 362.99 | 3.60 | 0.0870 |  |
| Residual | 1008.47 | 10 | 100.85 |  |  |  |
| Lack of Fit | 626.97 | 5 | 125.39 | 1.64 | 0.2995 | not significant |
| Pure Error | 381.50 | 5 | 76.30 |  |  |  |
| Cor Total | 10789.00 | 19 |  |  |  |  |

**Table S.10**

ANOVA Table for **Azithromycin**

| ANOVA for Response Surface Quadratic Model ***ER% Azithromycin*** | | | | | | |
| --- | --- | --- | --- | --- | --- | --- |
| Analysis of variance table [Partial sum of squares - Type III] | | | | | | |
|  | Sum of |  | Mean | F | p-value |  |
| Source | Squares | df | Square | Value | Prob > F |  |
| Model | 9941.25 | 3 | 3313.75 | 11.87 | 0.0002 | significant |
| A-pH | 912.55 | 1 | 912.55 | 3.27 | 0.0894 |  |
| B-volume of solvent | 2432.68 | 1 | 2432.68 | 8.72 | 0.0094 |  |
| A^2 | 6596.02 | 1 | 6596.02 | 23.64 | 0.0002 |  |
| Residual | 4464.95 | 16 | 279.06 |  |  |  |
| Lack of Fit | 3465.61 | 11 | 315.06 | 1.58 | 0.3218 | not significant |
| Pure Error | 999.33 | 5 | 199.87 |  |  |  |
| Cor Total | 14406.20 | 19 |  |  |  |  |

**Table S.11**

The quadratic polynomial models for nine responses and statistical parameters obtained from ANOVA tables for Central Composite Design (CCD)

| Responses | Final Equation in Terms of Coded Factors: | R-Squared | Adjusted R2 | Pred. R-Squared | Model  p-value | %  C.V | Adequate  Precision | Lack of Fit  p-value |
| --- | --- | --- | --- | --- | --- | --- | --- | --- |
| ER%  Amoxicillin | Ln(ER% amoxicillin) =  +3.33  -0.78 * A  +0.013 * B  +0.15 * C  +0.16 * B * C  -0.094 * B^2  +0.19 * C^2 | 0.9792 | 0.9696 | 0.9328 | < 0.0001 | 3.69 | 35.550 | 0.3419  not significant |
| ER %  Penicillin | ER% Penicillin =  +63.71  -24.70 * A  -2.57 * B  -1.78 * C  -7.87 * A * B  -3.12 * A * C  +6.38 * B * C  -4.55 * A^2  -9.68 * B^2  -4.38 * C^2  -4.13 * A * B * C  -2.34 * A^2 * C  +4.69 * B^3 | 0.9926 | 0.9798 | 0.9133 | < 0.0001 | 6.99 | 28.906 | 0.5758  not significant |
| _ER%_  _Cephalexin_ | ER cephalexin =  +54.05  -25.82 * A  +4.20 * B  +1.00 * C  -2.00 * A * B  -5.25 * A * C  +2.25 * B * C  +1.09 * A^2  -7.57 * B^2  -1.56 * C^2 | 0.9170 | 0.8424 | 0.7323 | 0.0003 | 20.12 | 12.899 | 0.8333  not significant |
| ER%  Ceftriaxone | ER ceftriaxone =  +61.78  -17.39 * A  +15.31 * B  -2.47 * C  -6.75 * A * B  +4.50 * A * C  +5.00 * B * C  -0.25 * A^2  -9.79 * B^2  -5.02 * C^2 | 0.9065 | 0.8224 | 0.5052 | 0.0252 | 19.50 | 11.548 | 0.2995  not significant |
| ER %  cefixime | ER cefixime =  +64.17  -17.94 * A  +15.34 * B  -2.86 * C  -8.12 * A * B  +6.63 * A * C  -9.68 * B^2  -5.44 * C^2 | 0.9073 | 0.8533 | 0.6138 | < 0.0001 | 17.36 | 15.045 | 0.2168  not significant |
| ER%  Ciprofloxacin | Ln(ER ciprofloxacin) =  +1.70  +0.36 * A  +0.81 * A^2 | 0.3514 | 0.2751 | 0.1464 | 0.0252 | 49.34 | 6.718 | 0.1827  not significant |
| ER%  Azithromycin | ER (%) azithromycin =  +39.22  -8.17 * A  +13.35 * B  +21.20 * A^2 | 0.70 | 0.64 | 0.50 | 0.0002 | 31.11 | 12.871 | 0.3218  not significant |
| ER%  Erythromycin | ER (%) Erythromycin =  +38.04  -7.39 * A  +12.72 * B  +23.74 * A^2 | 0.7026 | 0.6468 | 0.4481 | 0.0002 | 31.77 | 13.100 | 0.3287  not significant |
| ER%  Metronidazole | ER (%) Metronidazole =  +36.14  -7.17 * A  +12.26 * B  +23.15 * A^2 | 0.7261 | 0.6748 | 0.4798 | < 0.0001 | 30.45 | 13.872 | 0.1482  not significant |

The model terms A,B and C are coded values for indipendent variables, pH ,the amount of Na4EDTA and the volume of solvent respectivly.

**Table S.12**

Five proposed models for analysis of investigated antibiotics by multi-residues method

| No. | pH | Volume of  elution solvent | Na4EDTA | ER %  Amoxicillin | ER %  Penicillin | ER%  azithromycin | ER %  cefixime | ER %  Cephalexin | ER %  ciprofloxacin | ER %  Erythromycin | ER %  Metronidazole | ER %  ceftriaxone | Desirability |  |
| --- | --- | --- | --- | --- | --- | --- | --- | --- | --- | --- | --- | --- | --- | --- |
| 1 | 3.00 | 6.00 | 74.79 | 93 | 89.2915 | 81.9446 | 81.1395 | 86.4715 | 13.8007 | 81.8926 | 78.7183 | 84.3076 | 0.999 | Selected |
| 2 | 3.00 | 6.00 | 74.42 | 92.0723 | 89.2785 | 81.9448 | 81.4361 | 86.3921 | 13.6681 | 81.8929 | 78.7185 | 84.4818 | 0.997 |  |
| 3 | 3.04 | 6.00 | 75.00 | 92.4426 | 88.9033 | 81.1855 | 80.6733 | 85.985 | 13.6543 | 81.0691 | 77.916 | 83.9166 | 0.996 |  |
| 4 | 3.00 | 6.00 | 74.04 | 91.1199 | 89.2629 | 81.9448 | 81.7435 | 86.3081 | 13.5308 | 81.8928 | 78.7185 | 84.6614 | 0.995 |  |
| 5 | 3.00 | 5.94 | 74.78 | 93 | 89.001 | 81.5655 | 81.0192 | 86.643 | 13.9519 | 81.53 | 78.3687 | 84.0935 | 0.995 |  |

| 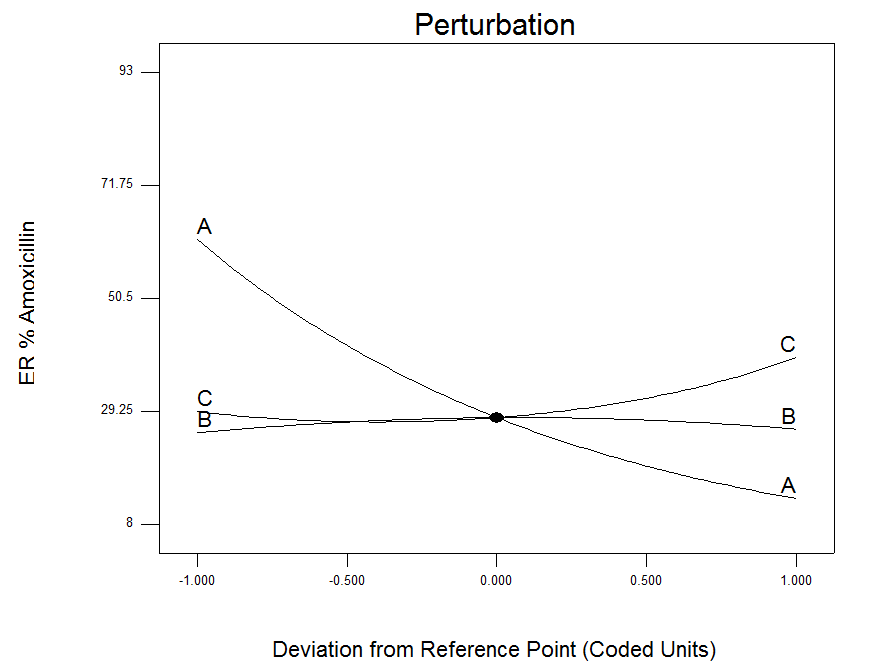 | 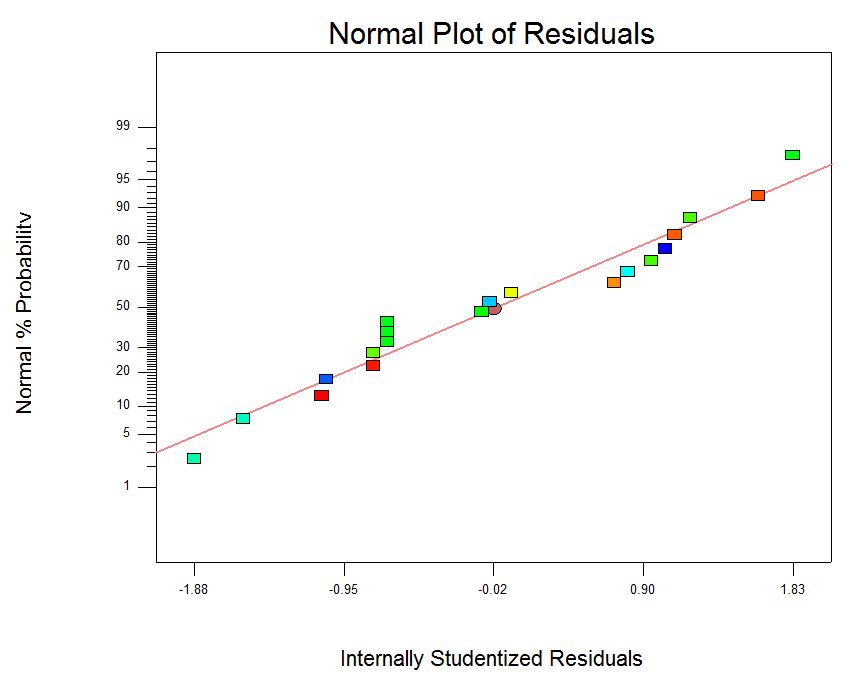 |
| --- | --- |
| (A) Perturbation plot for ER% Amoxicillin | (B) normality plot for ER % Amoxicillin |
| 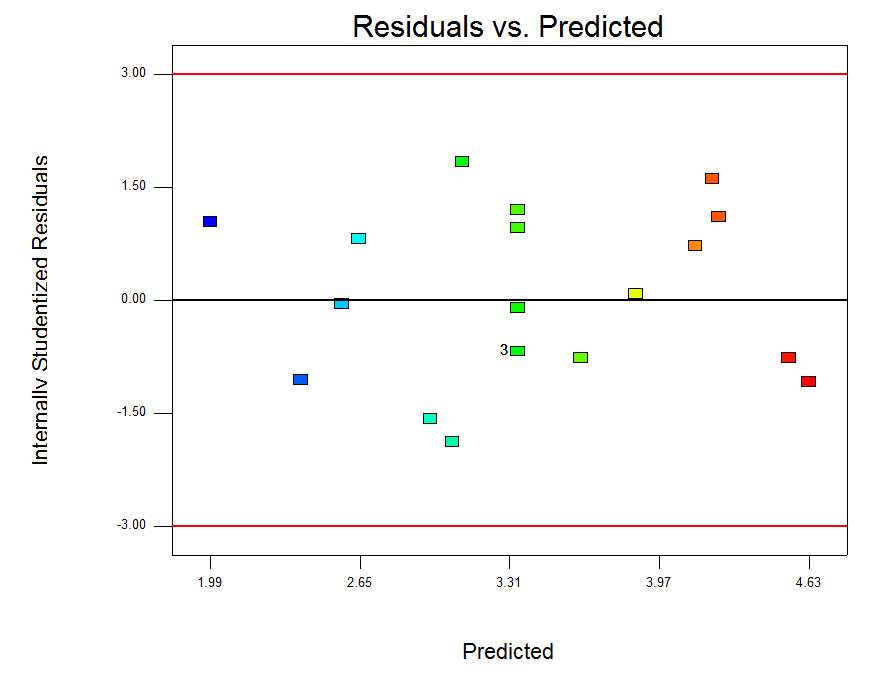 | *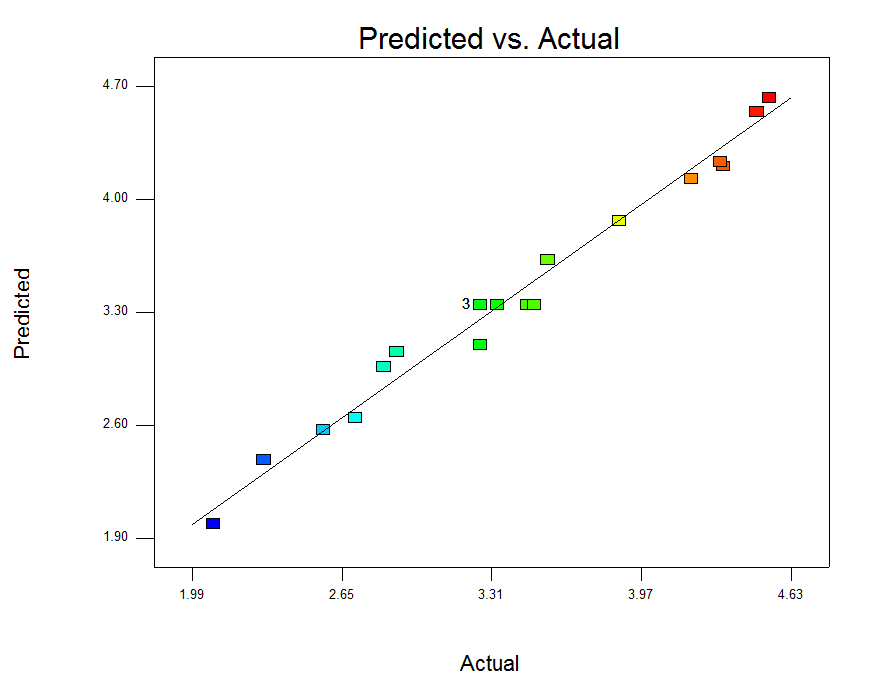* |
| (C) Internally studentized plot for ER% Amoxicillin | (D) Predicted vs. Actual plot for ER% Amoxicillin |

**Fig. S. 1**

Diagnostic plots for ER% Amoxicillin including: (A) Perturbation plot for ER% Amoxicillin, (B) normality plot for ER % Amoxicillin, (C) Internally studentized for ER% Amoxicillin, (D) Predicted vs. Actual for ER% Amoxicillin

| 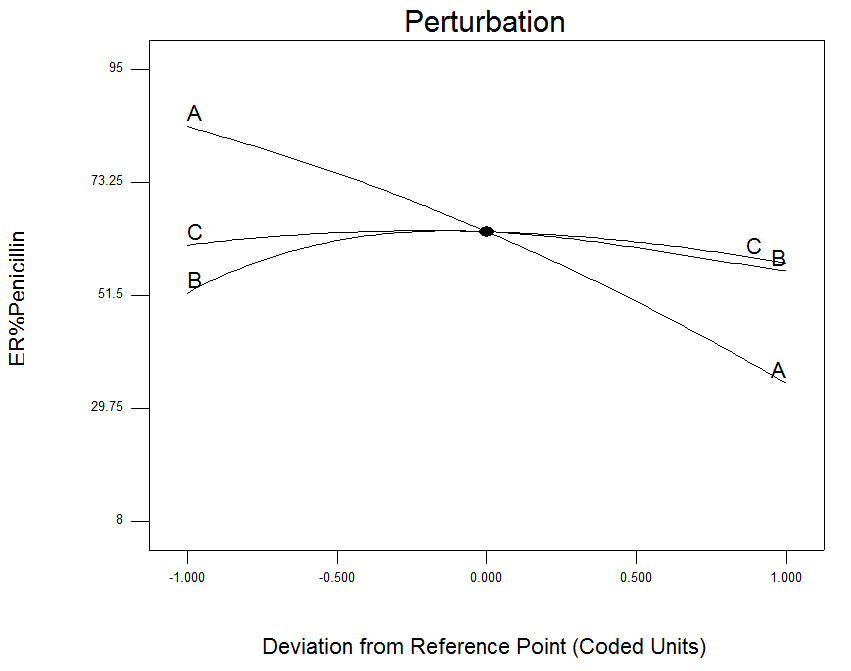 | 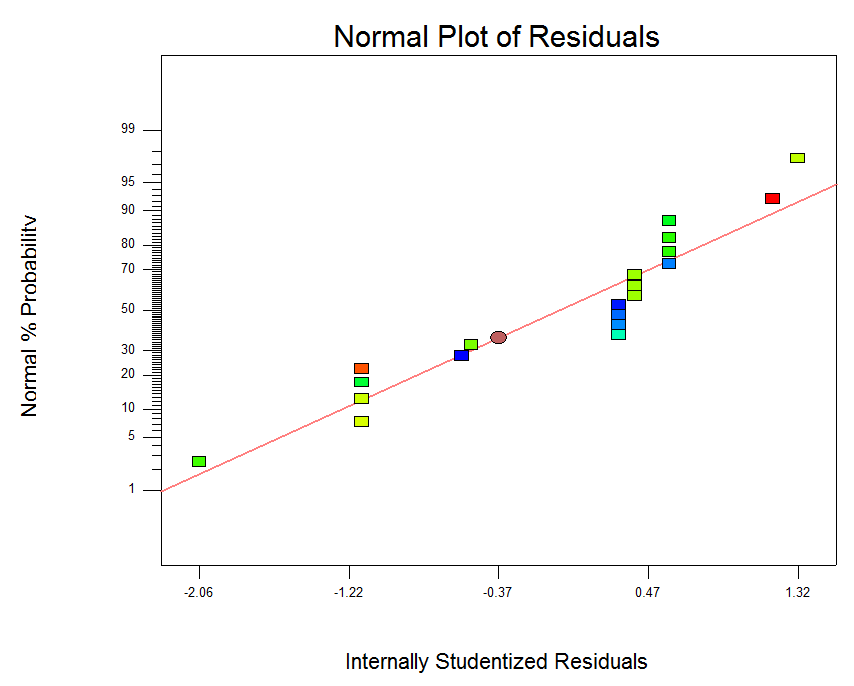 |
| --- | --- |
| (A) Perturbation plot for ER% Penicillin | (B) normality plot for ER % Penicillin |
| 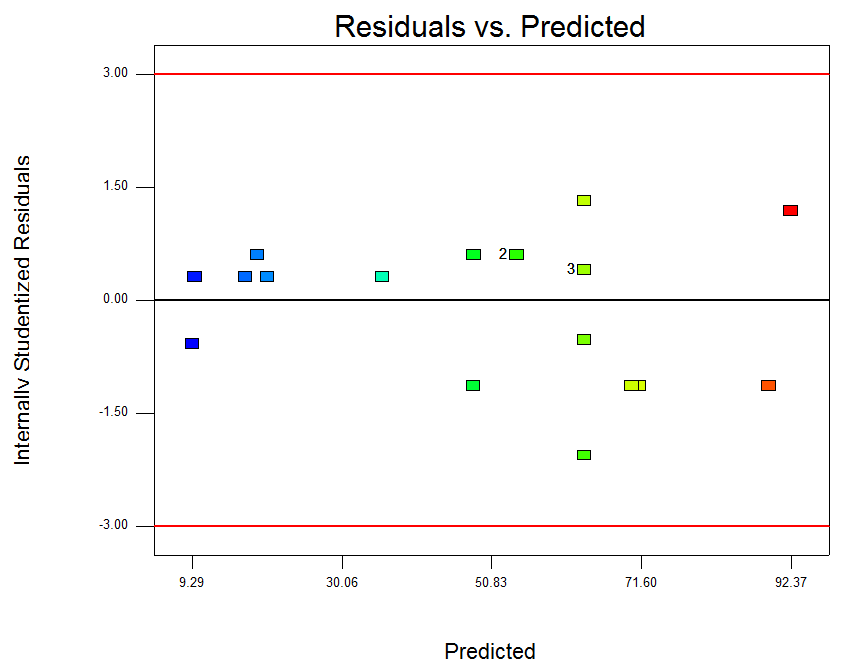 | 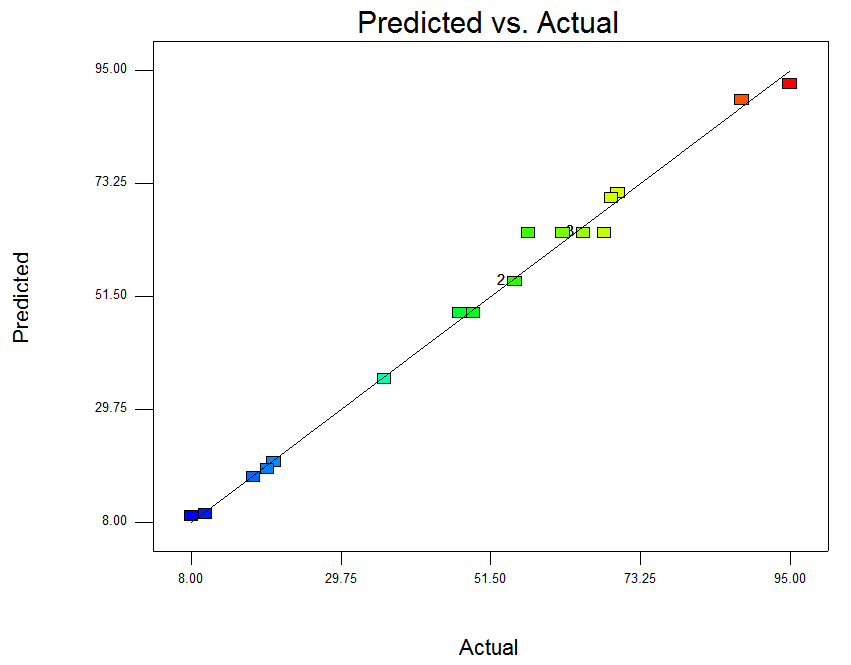 |
| (C) Internally studentized for ER% Penicillin | (D) Predicted vs. Actual for ER% Penicillin |

**Fig. S. 2**

Diagnostic plots for ER% Penicillin including: (A) Perturbation plot for ER% Penicillin, (B) normality plot for ER % Penicillin, (C) Internally studentized for ER% Penicillin, (D) Predicted vs. Actual for ER% Penicillin

**Fig. S. 3**

The well-separated chromatograms for MRM by the Zorbax-eclipse XDB-C18 column
